# The role of multidisciplinary diagnostic and therapeutic model of care in Lamb-Shaffer syndrome - case report

**DOI:** 10.1007/s13353-024-00838-3

**Published:** 2024-02-10

**Authors:** Urszula Sajewicz-Radtke, Ariadna Łada-Maśko, Małgorzata Lipowska, Bartosz M. Radtke, Beata S. Lipska-Ziętkiewicz, Katarzyna Krempla-Patron

**Affiliations:** 1 Laboratory of Psychological and Educational Tests, Gdańsk, Poland; 2 Institute of Psychology, University of Gdańsk, Bażyńskiego 8 Street, 80-309 Gdańsk, Poland; 3 Department of Biology and Medical Genetics, Medical University of Gdańsk, Gdańsk, Poland; 4 Neurologopedic Therapy Center, Gdańsk, Poland

**Keywords:** Rare genetic diseases, Intelligence, Language, Primary school, LAMSHF

## Abstract

This case study illustrates a multidisciplinary diagnostic and therapeutic model of care for a 7-year-old male with Lamb–Shaffer syndrome (LAMSHF). LAMSHF is an ultra-rare genetic neurodevelopmental disorder, caused by heterozygous alterations in the *SOX5* gene. An integrative model of therapy of cognitive functions and speech is described. The presented approach allows the development of language competences through stimulation of basic cognitive functions, which allows the learning of the abstract rules of an inflected language. A surprising, unexpected improvement in the cognitive functioning of the child was observed (both in terms of reasoning and speech), as well as an increase in his independence. The clinically important problem of the need for continued stimulation of cognitive development, in spite of the unfavourable prognosis associated with LAMSHF, is highlighted.

**Supplementary Information:**

The online version contains supplementary material available at 10.1007/s13353-024-00838-3.

## Introduction

Lamb–Shaffer syndrome (LAMSHF; ORPHA:530,983) is an ultra-rare genetic neurodevelopmental disorder, reported in less than 100 patients worldwide (Fukushi et al. 2018; Innella et al. 2021; Lamb et al. 2012; Zawerton et al. 2020). The disease is caused by haplo-insufficiency of the *SOX5* gene—that is to say, the functional lack of one of the two copies of the gene due to either a 12p12.1 microdeletion encompassing the gene *locus* or presence of variants in the gene sequence: either truncating variants or missense mutations affecting functional domains of the protein (Lamb et al. 2012; Zawerton et al. 2020). LAMSHF is most often caused by de novo deletions; however, in rare cases, it could be also inherited from parents when one parent is affected (Lamb et al. 2012; Zawerton et al. 2020). *SOX5* encodes a transcription factor involved in the development of the nervous system and occasionally in other developmental processes (Kamachi & Kondoh 2013; Zawerton et al. 2020).

The LAMSHF clinical spectrum is very wide and remains understudied. The majority of patients present with global developmental delay and speech delay (Fukushi et al. 2018). Moreover, different degrees of intellectual disability are observed in LAMSHF patients (Innella et al. 2021). Several dysmorphic facial features, such as frontal bossing, depressed nasal bridge, epicanthal folds, short philtrum, crowded teeth, auricular abnormalities and skeletal abnormalities are also common (Zawerton et al. 2020). Furthermore, behavioural problems are observed—most commonly autism spectrum disorder, anxiety, tantrums, aggression and hyperactivity (Fukushi et al. 2018; Innella et al. 2021; Zawerton et al. 2020).

This report details the clinical and therapeutic characteristics of a 7-year-old Polish male with a de novo microdeletion at chromosome 12p12.3p12.1 encompassing 26 protein-coding genes, *SOX5* comprised. At the time the diagnosis was made, the patient presented delayed psychomotor development, movement clumsiness, aphasia, mild intellectual disability, microcephaly and facial dysmorphia.

## Background information

The patient is the only child of young, non-related parents of Polish origins. As of the writing of this paper, he lives with both his parents and is enrolled in the first grade of primary school. He is in a special needs education program, based on a diagnosis from a psychological-educational counselling centre (dual diagnosis: aphasia and intellectual disability).

### Pregnancy and childbirth

The patient was born at term by caesarean section due to risk of eclampsia as the mother had elevated blood pressure in the third trimester; otherwise, the course of pregnancy was uneventful. The mother was on thyroid-hormone supplementation due to hypothyroidism diagnosed before pregnancy. He was born with a birth mass of 2910 g, APGAR scores 5 at 0′, 9 at 5′ and eventually 10 at 10′; he received antibiotics and 3 days of oxygen therapy in the neonatal period due to congenital infection.

### Early psychomotor development

The early psychomotor development of the patient was disharmonic. From 3 months of age, it was necessary to support his motor development with rehabilitation in an early intervention clinic. The patient started walking around 1.5 years old, before he learnt to crawl. It should be noted that the motor development of the patient was in general in the normal range—however, rehabilitation support from the early months of life was necessary. The patient said his first words around 1.5 years of age, and his first sentences when he was 4. From birth, he was also under the care of a neurologist. At the age of 2 years and 5 months, a brain MRI examination revealed abnormalities of gyration, especially in the frontal lobe. The patient was deemed disabled based on the diagnosis of aphasia and polymicrogyria. Because of the specific physiognomy of the patient and microcephaly, he underwent genetic examination, resulting in the diagnosis of Lamb–Shaffer syndrome. The medical report included a diagnosis of moderate intellectual disability, diagnosed without a psychological examination.

#### Functioning in preschool

The patient attended an integrated preschool 4 days a week for 4 h. The teachers reported the patient as having significant behavioural difficulties and that he was aggressive towards his peers. He was being administered *Risperidonium* prescribed by a psychiatrist. The teachers were not interested in a visit by the patient’s psychologist nor did they provide video recordings from the preschool. Importantly, the patient did not present behavioural difficulties in any of the classes he took outside the preschool.

## Assessment and diagnosis

**Fig. 1:**
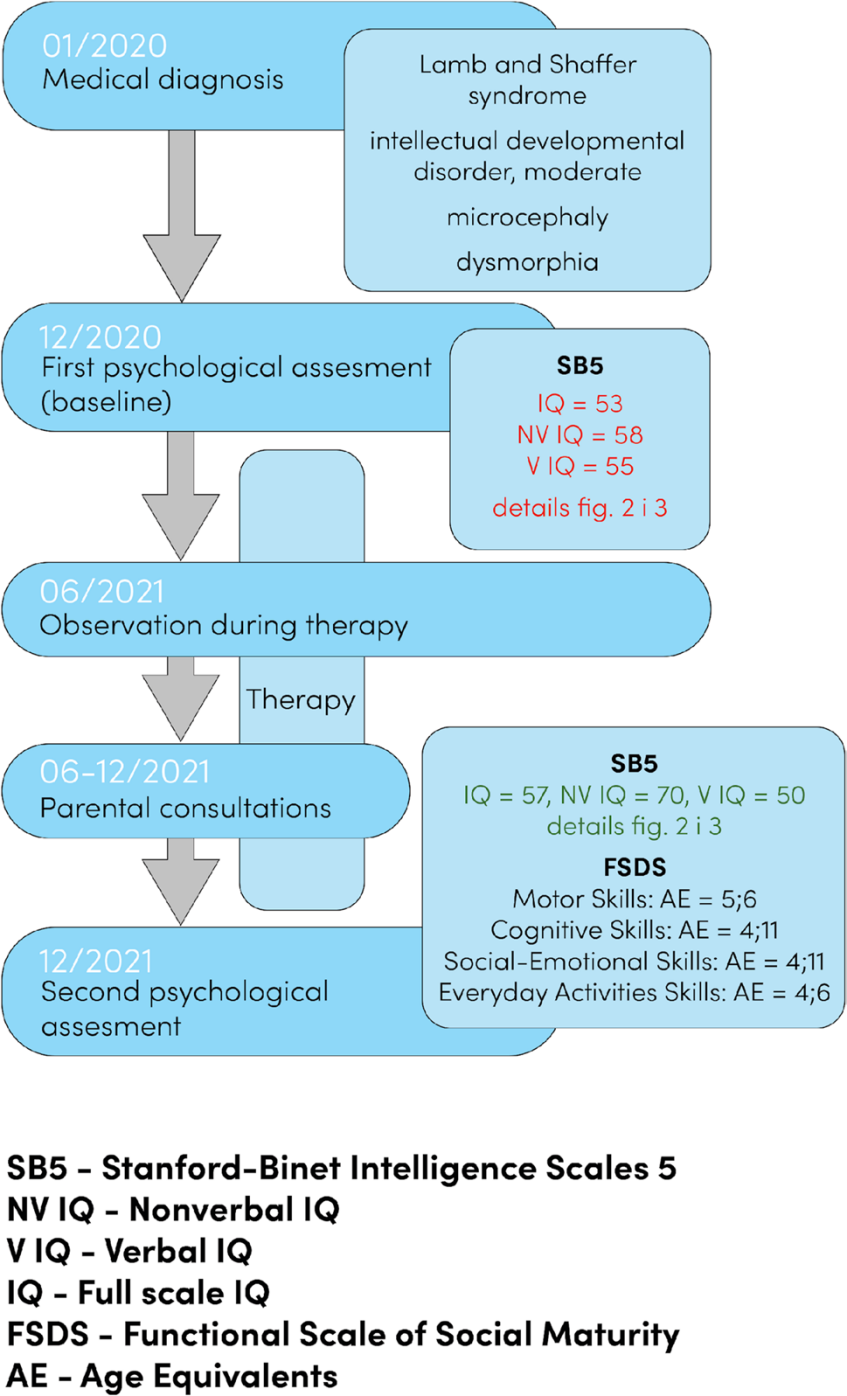
Diagnostic and therapeutic timeline

At the age of 5, the patient was brought by his parents to a specialist psychological–pedagogical clinic in order to assess his current levels of cognitive development and to plan further therapy (Fig. 1). After the initial psychological consultation, a psychological examination with the diagnostic tools presented in Table 1 was planned. The psychologist who performed the diagnosis did not take part in the therapy that followed, in order to prevent the diagnostic process from influencing the therapeutic process.

**Table 1:** Diagnostic tools

Diagnostic tool	Stanford-Binet Intelligence Scale—Fifth Edition (SB5)	Functional Scale of Social Maturity (FSDS)
Authors	Roid, (2003) Polish adaptation: Roid et al. (2017)	Sajewicz-Radtke and Radtke (2023)
Age range	2;0–69;11	1;0–30;00
Application	− Assessment of intelligence and cognitive abilities, also in patients with special needs (e.g., learning difficulties, autism spectrum disorder, intellectual disability or rare genetic diseases) − SB5 was selected because despite the delayed development of the patient’s speech, it seemed best to use a method with a wide spectrum of measurement of the structure of intelligence, in order to appropriately select intervention methods	− Individual diagnostics of persons who present difficulties in adaptation and independent functioning in certain conditions (e.g., at school, home, work) − Complementary to the process of diagnosing the level of intellectual disability, in that it allows the assessment of the level of functioning in relation to IQ measured using the SB5
Scales and subscales	Ten subscales—five nonverbal and five verbal. Both nonverbal and verbal subscales refer to each of the following cognitive factors: − Fluid reasoning − Knowledge − Quantitative reasoning − Visual-spatial processing − Working memory	Four areas and 16 subscales: − Motor skills—subscales: (1a) gross motor skills, (1b) fine motor skills − Cognitive skills—subscales: (2a) speech development, (2b) speech comprehension, (2c) concepts − Social-emotional skills—subscales: (3a) social relationships, (3b) self-regulation and self-steering, (3c) free time − Everyday activities skills—subscales: (4a) food, (4b) getting dressed, (4c) using the toilet, (4d) hygiene, (4e) everyday life and independence, (4f) health and responsibility, (4 g) money, (4 h) school and work − Allows the calculation of developmental age (for overall functioning of the patient and for each of the subscales and areas) and the functioning quotient (the so-called Doll’s index)
Assessment duration	45–90 min	60 min
Reliability	Assessed using the omega factor − full IQ—0.98 − nonverbal IQ—0.95 − verbal IQ 0.96	Assessed using Cronbach’s alpha: − Motor Skills—0.97 − Cognitive Skill—0.98 − Social-Emotional Skills—0.98 − Everyday Activities Skills—0.99 − General score—0.99

### Psychological assessment

#### First psychological assessment—baseline

The assessment took place in December 2020. At that time, the patient was 5 years and 10 months old.

The overall intellectual ability measured by the Stanford-Binet 5 Intelligence Scale (Roid 2003; Roid et al. 2018) was at the level of mild intellectual disability [general IQ 53 (lower CI = 50; upper CI = 60); nonverbal IQ 58 (lower CI = 54; upper CI = 67); verbal IQ 55 (lower CI = 51; upper CI = 65)]. The breakdown of the results achieved by the patient indicated that the development of intellectual functions was disharmonic in the nonverbal and the verbal dimensions. No statistically significant difference between verbal and nonverbal abilities was observed, which is why, despite a significant delay in speech development, a decision was made to use the general score for interpretation. In that period, the intellectual functioning of the patient was on the level of a 3-year-old child. Because the degree of impairment of speech development was significantly greater than what would be expected of a patient with mild intellectual disability, a co-diagnosis of intellectual disability and developmental aphasia was made.

#### Observation during therapy

In June 2021, the patient was observed in a therapeutic class in order to assess his progress and identify potential adjustments to the therapy intervention. The patient still exhibited poor motor coordination. It was observed that he may have trouble with his eyesight, and there was a need to enlarge the therapeutic materials, because the patient was complaining that he could not see them properly. The patient had significantly less trouble with inflection. During the sessions, he was eagerly interacting with both the therapist and the observing psychologist. He was trying to make jokes and include the therapist in his play. However, he had significant troubles with “why?” and “what?” questions. He did not understand the intentions of the person asking, and he did not use hints or help. He also had trouble with joint attention and kept on asking the same questions. He was extremely impulsive, and he had significant difficulty with inhibiting his reactions.

#### Consultations with parents

Up to December 2021, 12 parents’ consultations were held. During that period, a significant improvement in the father–son bond, an increase in the patient’s independence in terms of self-care activities and greater involvement of the patient in everyday family life (including him in daily duties and chores) were observed.

#### Second psychological assessment

The second psychological assessment took place in December 2021, when the patient was 6 years and 11 months old. In comparison with the previous assessment, the patient was more capable of holding a conversation and preferred topics that were of interest to him. In comparison to the previous assessment, the patient made better use of hints and help; he needed fewer demonstrations and repetitions in order to learn a new skill. He still exhibited serious trouble with planning his work and often used the trial-and-error approach. The patient was able to make four-element patterns with help; however, his levels of visual analysis and synthesis are worrying in the context of reading and writing.

The overall intellectual ability of the patient measured by the Stanford-Binet 5 scale (Roid 2003; Roid et al. 2018) was at the level of mild intellectual disability [IQ = 57(lower CI = 54; upper CI = 63)]. The statistically significant difference between the nonverbal (IQ = 70 (lower CI = 65; upper CI = 79)) and verbal (IQ = 50 (lower CI = 46; upper CI = 60)) scales was notable, with the non-verbal dimension being at an advantage.

The patient’s intellectual development in the non-verbal dimension was on the border of intellectual disability (still within the norm), while in the verbal dimension, it was at the level of mild intellectual disability. This is a significant change in comparison with the previous examination.

**Fig. 2:**
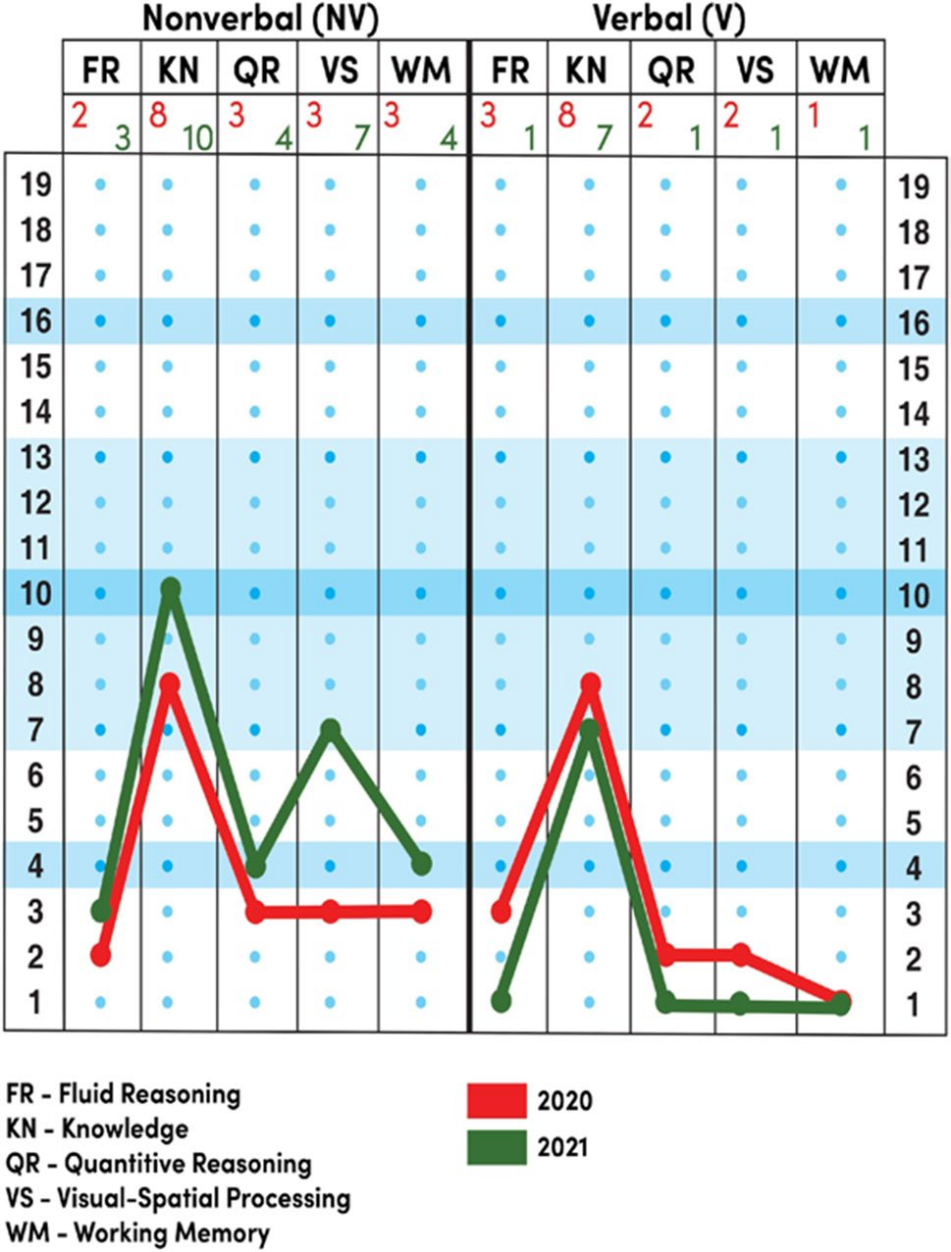
Profilogram of the recalculated results—comparison of first and second intelligence assessments

**Fig. 3:**
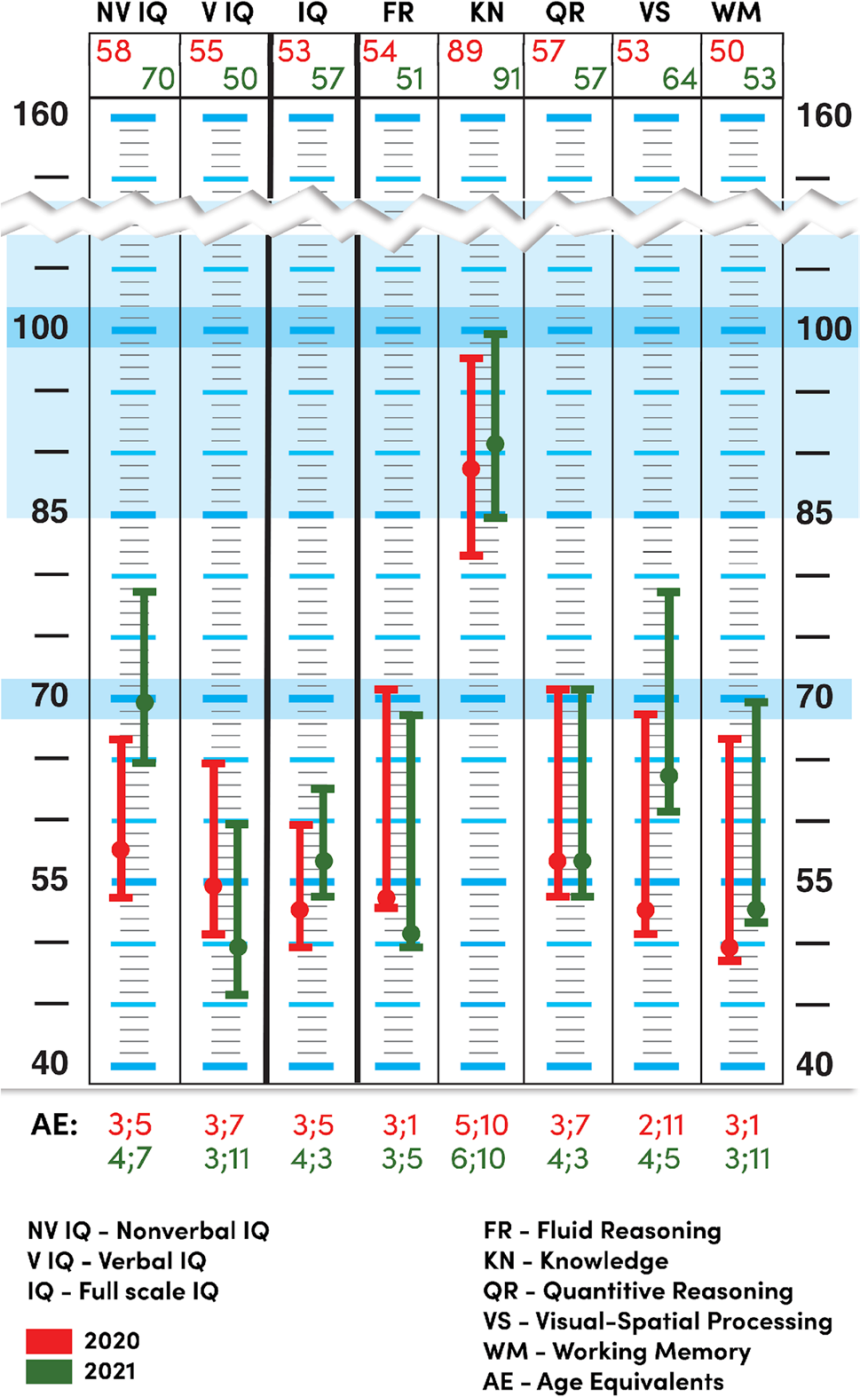
Psychogram—comparison of the first and second intelligence assessments

Analysis of the psychogram indicates that the patient exhibited serious difficulties with fluid reasoning. The ability to solve logical tasks based on discovering relationships between different objects was developing on a low level. Neither verbal instructions nor demonstrations helped him understand the essence of a task. In this, the patient’s results did not differ from those observed a year prior (Figs. 2 and 3). The mental age of the patient in relation to this subscale was assessed at 3;5 years.

The level of general knowledge presented by the patient, gathered through both formal education and socio-cultural stimulation, is indicative of appropriate stimulation for development. This metric indicated correct levels of procedural knowledge and general knowledge about the world. The scope of his knowledge and intellectual interests was the patient’s strongest suit. Despite serious language deficits, the patient tried to define words, mainly through how a given thing is used. On this scale, the patient’s mental age is on the level of his actual age, and his IQ on the Knowledge subscale is 91. This may also indicate appropriate selection of educational and therapy tools.

The assessment of quantitative reasoning, understood as the ability to solve problems and mathematical tasks, is developing on a low level. This result indicates severe difficulties understanding the concept of numbers, estimating and/or solving problems and making measurements. The patient also exhibited serious difficulties in terms of even simple counting. He had difficulties understanding the abstract concept of how numbers are written—he dealt better with concrete material. On this scale, the mental age of the patient was at the level of 4;3 years.

He performed visuospatial tasks more poorly than do his peers. He had significant difficulties with identifying patterns and relationships in visual material. His spatial orientation skills were low, as was his ability to understand concepts associated with this domain. This factor was better developed in the nonverbal area, which requires less use of language. The patient had significant difficulties with integrating visual stimuli, which may significantly impact the speed at which he will acquire reading skills. Importantly, after initiating visual therapy classes, a significant improvement in scores for nonverbal visuospatial processing was observed (2020—calculated score, 3; 2021—calculated score, 7).

The patient’s working memory was at a low level. This indicates significant difficulties in developing this category of memory processes, in which information contained in short-term memory is checked and grouped, and operations are performed on it. In this domain, the mental age of the patient was around 3;11.

In order to assess the patient’s adaptive behaviours, the Functional Scale of Social Maturity (Sajewicz-Radtke & Radtke 2023) was used. In the domain of motor development, the patient scored low in terms of fine motor skills (it can be observed that his hands do not have typical dexterity, although they allow for full independence even with low precision of movements). In terms of the gross motor skills, despite his clumsiness, the patient achieved all the milestones for his age. In terms of speech and communication, the patient scored low on speech development, speech understanding and concepts. This is associated with his aphasia diagnosis. His speech was comprehensible to others: he can make small purchases, ask for help, etc. However, this requires the goodwill of his environment. Nonetheless, the patient had trouble with understanding symbolic statements; he had difficulties with the concept of time: he not only did not understand the clock, but also did not understand concepts such as “in some time” and “right after”. The patient also scored low on the scale of social development. He had trouble controlling his behaviour. The patient had trouble understanding and regulating his own emotions. The patient scored low in the practical sphere. He could eat meals on his own, but very clumsily due to getting lost in thought and not being able to direct the food into his mouth. He was not fully able to dress himself yet; he made attempts to do so, and he was very determined in these attempts, but he still required the support of an adult, especially when in a hurry. He used the toilet on his own, and he signalled the need to do so himself. He did not take care of his hygiene on his own; he must always be reminded about the need to wash his hands or his teeth. He needed support when using money. This is an area of intensive natural-environment training: in the local shop, cashiers trained by a psychologist support the patient’s attempts to use money. However, he was still unable to count the right amount of money and he did not always know whether he had enough money. When working one-on-one, he was good at cooperating and listened to feedback and behavioural corrections. When working in a group at the kindergarten, he was somewhat prone to impulsive behaviours and lack of cooperation.

#### Speech assessment

The patient communicates verbally using complex sentences. He asked his interlocutors questions, but he had difficulties giving answers. He often answered questions based on his experiences or things/situations that he associated with the given topic. It was difficult to get precise answers to questions out of the patient.

In terms of language programming, the patient had a significantly higher active vocabulary of nouns than verbs. Despite the acquired skill of building sentences, due to insufficient knowledge of verbs, the patient often built sentences that did not precisely communicate his needs. The patient had only mastered adjectives as oppositions (e.g. big–small), and only regarding basic characteristics. This skill was usually not used by him in practice, because if an adjective was presented to the patient separately from the opposite word, he was unable to use it. In the case of noun-derived adjectives, the patient usually correctly presented names. His difficulties with learning distinct parts of speech also affected prepositions. The patient, despite a good knowledge of the prepositions “on” (Polish: “na”) and “under” (Polish: “pod”), was unable to differentiate between them (even when working with concrete material). In terms of inflection, the patient usually used the noun declension correctly in the present tense for the following cases: genitive, accusative, instrumental and locative. The use of dative in the present tense was still problematic. More difficult grammatical constructions, such as adverbial sentences (causes), which are necessary in the process of argumentation, were not used by the patient at the time of this evaluation. The patient also had difficulty building sentences in the appropriate tense. When the child was asked what he did today, he answered in the present tense. The use of future tense also comes with difficulty—both in linguistic and cognitive terms. These deficits influenced the comprehensibility of the content of what the patient was trying to communicate.

Apart from language difficulties, the patient also presented deficits in terms of the distribution of muscle tension in the stomatognathic complex. The patient presented incorrect placement of the tongue at rest. Currently, the patient works with a speech–language pathologist in order to improve breathing and desensitize the area beneath the tongue. The patient had also undergone a consultation with an orthodontist and qualified for treatment using a bioblock orthodontic appliance. Moreover, a laryngologist diagnosed the patient with nasal septum deviation (which is to be corrected after the main period of growth). The medical difficulties detailed above affect the patient’s muscular abilities.

### Therapy course

The patient started his speech therapy at the age of 2;5 years. The initial state of communication and language did not allow him to establish rapport with the environment. The child did not have basic communication skills, such as eye contact, mimicry and turn taking. However, the patient presented nonverbal communication behaviours, such as crying, pointing at an item that interests him with his eyes, smiling and jumping in his chair. Table 2 describes the stages of the patient’s speech therapy.

**Table 2:** Stages of the patient’s speech therapy

Stage of therapy	Main areas	Intervention	Results	Observed difficulties
First	Basic communication functions	- Frequency of classes: 2 h a week Learning: - Cooperation with the therapist - Collaboration; - Mimicry in play - Alternating when playing - Eye contact	- Building rapport with the therapist and getting ready for the next stage—verbal communication training	- Building rapport - Previous negative experiences with therapy
Second	Articulating first speech sounds	- Manual stimulation of speech sounds - Mimicking the positioning of organs of articulation and thus learning speech production	- Building a full rapport with the therapist - Using help and asking for help when positioning the organs of articulation - Readiness to learn the grammar of his native language	- Mimicking speech sounds - Remembering movement sequences - Remembering vowels, syllables and words
Third	Learning language and comprehension	- Learning using concrete material (instead of photos, images and other abstract materials, everyday use items are used). Cognitive functions trained: the constancy of an item, precise labelling - Making linguistic categorisations together with learning the parts of speech. Cognitive functions trained: selecting important clues, spontaneous comparison - Learning to understand analogies, using the Instrumental Enrichment Basic cognitive function training (Ben-Hur & Feuerstein 2011; Salas et al. 2010). Cognitive functions trained: spontaneous comparison, making and verifying hypotheses - Practical learning of parts of speech, such as the declension of nouns, and declensions and prefixes of verbs, while performing concrete activities. Cognitive functions trained: recognizing and understanding the relationships between elements - Making categories of “noun declension” for each case separately - Differentiation between categories of distinct cases in noun declension - Introduction of personal pronouns - Making categories of “verb declension” - Differentiation between the categories of verb declension	- Associating speech sounds with concrete objects - Transfer of the skills learned during practical training to other activities, not previously trained (generalising skills to everyday conversation, work cards) - Mastering oppositional and noun-based adjectives	- No effects when using standard language exercises - Remembering the order of words in a sentence - Learning grammar rules (mostly the inflection of the native language) - Learning exceptions to inflection rules in the native language - Not seeing analogies, resulting in problems with seeing rules governing the language, i.e., identifying them in a stream of speech, remembering them and then using them in practice - Difficulties with visual processing (no specific diagnosis) - Problems with telling apart perfective and imperfective verbs - Problems with temporal orientation - Adjective gradation - Learning spatial relationships

At the time of the second assessment, the patient could communicate fluently using sentences (in present, past and future tenses), ask questions and take part in dialogue. Development of his linguistic and cognitive processes was supported during the Instrumental Enrichment Basic classes. These classes combine cognitive and language exercises that are appropriate for the patient’s continued developmental needs. In order to do so, the therapists focus on stimulating the following functions: auditory and visual synthesis and analysis, categorising and understanding relations and dependencies.

## Discussion

The applied cognitive function training forms a basis on which the speech therapist can build an understanding of abstract linguistic rules. Based on our clinical practice, we can establish with some certainty that the learning of an inflectional language by children with rare genetic disorders requires intensive, focused and integrated training of cognitive functions. Because it is difficult to establish the direction of the language–thought relationship, it is necessary to combine these two therapeutic areas into one integral therapeutic process (Mahon & Kemmerer 2020).

Previous studies regarding the functioning of patients with Lamb–Schaffer syndrome (LAMSHF) indicate that patients score at the levels of mild or moderate disability in terms of their IQ (Innella et al. 2021; Lamb et al. 2012), whereas our patient exhibited a surprising, statistically significant increase in the IQ score in the nonverbal area relative to other children with LAMSHF. It was not expected that the results on the nonverbal scale would reach the level of intellectual norm. The increase of the scores in NVK (non-verbal knowledge) and NVVS (non-verbal visual-spatial processing) probably stems from the specifics of the program used to support the patient’s cognitive development. Instrumental Enrichment Basic significantly focuses on stimulating cognitive curiosity and understanding the relationships between facts and/or events (Salas et al. 2010). Both subscales in which increased scores were observed require the patient to use these skills. The results on the Knowledge subscale, which are adequate for the patient’s age, suggest that the designed therapy program suits his cognitive needs. This factor is an indicator of general knowledge, gathered both in formal education and as a result of socio-cultural stimulation, and it is indicative of adequate stimulation of cognitive development (Roid 2003; Roid et al. 2018). The observed results support the validity of including Instrumental Enrichment Basic in the patient’s therapy. Further interventions should concentrate on stimulating fluid reasoning and numerical reasoning, which would help increase the patient’s independence, for example in the use of money.

## Limitations and strengths

The present study provides very valuable results for scientists, but importantly also for practitioners who work every day with children with rare genetic diseases. Despite the fact that our study is a case study report and this limits the generalizability of the results as well as the heterogeneity of the clinical spectrum in Lamb–Schaffer syndrome, the unexpected effects of cognitive function therapy we obtained are worth considering for implementation in other patients with LAMSHF.

The findings of our study indicate that it is necessary to include cognitive stimulation programs in the therapy processes of people with genetic syndromes, even if previous reports indicate a low level of cognitive functioning in a particular syndrome (Innella et al. 2021; Lamb et al. 2012; Zawerton et al. 2020). This can increase the quality of life of patients and their families as well as increase patients’ independence.

Our research also suggests that a multidisciplinary, holistic approach (including, e.g., geneticists, psychologists, speech therapists, physiotherapists, optometrists and the patient’s parents) to working with children with rare genetic diseases enables the best possible development opportunities despite the difficulties experienced. In our case, the cooperation of the speech–language pathologist and the psychologist, who assessed the patient’s intellectual progress and supported developing the patient’s language system, turned out to be crucial. Moreover, due to the limitations related to vision problems, it will be important to include an optometrist in the group of specialists dealing with the patient going forward, which can significantly increase the possibilities for future therapy.

However, the results of our research should be also considered in the context of several limitations. The most important is that the quality of the current study would be significantly increased had it used a randomized control study design implementing the proposed therapeutic approach involving more children with LAMSHF. Unfortunately, this is very difficult due to the fact that this is a very rare genetic disease. However, it is undoubtedly a very valuable direction for future research.

Further research should also assess the long-term effects of the proposed speech therapy combined with cognitive function training through a follow-up assessment. In the future, it is important to develop standards for working with children with rare genetic diseases, taking into account a holistic multi-specialist approach to their diagnosis and therapy.

## Supplementary Information

Below is the link to the electronic supplementary material.

Supplementary file1 (DOCX 13 KB)

MOESM2 (PDF 176 KB )

## Data Availability

The presented data in this study are available from the corresponding author upon request.
