# Supplementary material for: The role of multidisciplinary diagnostic and therapeutic model of care in Lamb-Shaffer syndrome - case report
Source: J Appl Genet. 2024 Feb 10;65(4):747–55. doi: 10.1007/s13353-024-00838-3 (PMC11560983; doi:10.1007/s13353-024-00838-3)
Supplement: Supplementary file 1 — Supplementary file1 (DOCX 13 KB) [file 13353_2024_838_MOESM1_ESM.docx]

# Appendix A

*A word from the patient’s parents*

We are the parents of a disabled child. Today we can say this outright, but at the beginning it was hard for us to see how special our son is. It is extremely difficult to assess the progress of the development of one’s own child, especially when the progress is made slowly and with small steps.

The difference in our son’s behaviour and cognition in comparison with his peers used to overwhelm us. However, thanks to professional support from therapists, we learned to see and to find joy in the small-great successes.

We have come a long way from empty stares, trouble getting his attention, and nonverbal communication to advanced conversation using full sentences, even for abstract topics. Every new activity, word, and spark of intelligence in our son’s eyes brings us comfort.

The life of our child and our lives look completely different thanks to the huge work that our little hero has done. He is a joyful, sensitive, and empathetic patient, and is great at communicating. With every ‘why?’ question, the small world of our family becomes bigger and bigger, more interesting, and more full of opportunities.
