# Supplementary material for: The role of multidisciplinary diagnostic and therapeutic model of care in Lamb-Shaffer syndrome - case report
Source: J Appl Genet. 2024 Feb 10;65(4):747–55. doi: 10.1007/s13353-024-00838-3 (PMC11560983; doi:10.1007/s13353-024-00838-3)
Supplement: Supplementary file 2 — (PDF 176 KB ) [file 13353_2024_838_MOESM2_ESM.pdf]

CARE Checklist (2013) of information to include when writing a case report

(cc) BY-NC-ND

Topic	Item	Checklist item description	Reported on Page
Title	1	The words "case report" should be in the title along with the area of focus	2
Key Words	2	2 to 5 key words that identify areas covered in this case report	2
Abstract	3a	Introduction—What is unique about this case? What does it add to the literature?	2
	3b	The main symptoms of the patient and the important clinical findings	2
	3c	The main diagnoses, therapeutics interventions, and outcomes	2
	3d	Conclusion—What are the main "take-away" lessons from this case?	2
Introduction	4	One or two paragraphs summarizing why this case is unique with references	2-3
Patient Information	5a	De-identified demographic information and other patient specific information	3
	5b	Main concerns and symptoms of the patient	3-4
	5c	Medical, family, and psychosocial history and genetic information or relevant comorbidities.	3-4
	5d	Relevant past interventions and their outcomes	3-4
Clinical Findings	6	Describe the relevant examination (e.g psychological) and other significant clinical findings.	5-17
Timeline	7	Important information from the patient's history organized as a timeline	3-5
Diagnostic Assessment	8a	Diagnostic methods (psychometric and clinical)	6-7, 13-14
	8b	Diagnostic challenges (such as communication restrictions, or cultural)	6, 7- 8, 13-14
	8c	Diagnostic reasoning including other diagnoses considered	3-4
	8d	Prognostic characteristics where applicable	-
Therapeutic Intervention	9a	Types of intervention (psychological, speech therapy, occupational, and others)	15-17
	9b	Administration of intervention (duration and frequency of meetings,)	15-17
	9c	Changes in intervention (with rationale)	-
Follow-up and Outcomes	10a	Clinician and patient-assessed outcomes (when appropriate)	-
	10b	Important follow-up diagnostic and other test results	7-13
	10c	Intervention adherence and tolerability (How was this assessed?)	-
	10d	Adverse and unanticipated events	-
Discussion	11a	Discussion of the strengths and limitations in your approach to this case	19-20
	11b	Discussion of the relevant literature.	18-19
	11c	The rationale for conclusions (including assessment of possible causes)	18-19
	11d	The primary "take-away" lessons of this case report	18
Patient Perspective	12	When appropriate the patient should share their perspective on the treatments they received	Apx A
Informed Consent	13	Did the patient give informed consent? Please provide if requested	Yes X No ☐
